# The role of primary physician training in improving regional standardized management of diabetes: a pre-post intervention study

**DOI:** 10.1186/s12875-022-01663-5

**Published:** 2022-03-21

**Authors:** Hanbing Liu, Huimin Hou, Mingfeng Yang, Yusheng Hou, Zhongyan Shan, Yanli Cao

**Affiliations:** Department of Endocrinology and Metabolism, Institute of Endocrinology, Liaoning Provincial Key Laboratory of Endocrine Diseases, The First Affiliated Hospital of China Medical University, China Medical University, Shenyang, Liaoning China

**Keywords:** Diabetes mellitus, Standardized management, Primary physician training, Complication screening

## Abstract

**Background:**

Hierarchical diagnosis and treatment has been gradually implemented throughout the China. Primary physicians are the main force in primary-level medical and health services, which means that standardized training of primary-level doctors is indispensable.

**Objectives:**

Evaluation of the effect of primary physician training on standardized management of diabetes, and comparison of the effects of different training models.

**Method:**

The study selected 24 community health service centers from 4 cities in Liaoning Province, and consisted of two groups: primary physicians (*n* = 2083) who received training; and patients with diabetes (*n* = 585) in community health service centers. Short-term training effects on primary physicians were assessed through diabetes knowledge tests at baseline and at the end of training; the long-term effects of training on patients with diabetes were assessed by questionnaires at baseline and 1 year after training. The differences in training effects between different training models were compared. Complication screening results were also assessed.

**Results:**

After training, the primary physicians’ knowledge of diabetes diagnosis and treatment improved (*p* < 0.05). The complication screening rate of local diabetes patients increased from 22.2% before training to 27.7% 1 year after training (*p* = 0.033). There were significant differences in the training effect between different training models (*p* = 0.038). The short-term intensive training group demonstrated the greatest training effect, primary physicians under this training model are more likely to conduct standardized screenings for patients (OR = 1.806, 95%CI 1.008–3.233), and the complication screening rate was the highest (37.6%).

**Conclusion:**

This study shows that training of primary physicians is an effective way to improve the standardized management of diabetes, by improving the ability of primary physicians to manage diabetes in a standardized manner, so that patients in primary hospitals receive more comprehensive diagnosis and treatment services. Compared with scattered training throughout the year, short-term intensive training was found to be more effective.

**Supplementary Information:**

The online version contains supplementary material available at 10.1186/s12875-022-01663-5.

## Background

The high incidence of diabetes and related disability and mortality has become a worldwide public health problem [1, 2]. As the world’s largest developing economy, China has gradually become the center of the global diabetes epidemic [3]. In China, the prevalence of diabetes has increased from 9.7% in 2010 to 10.4% in 2013, and to 11.2% in 2017, reaching 12.8% in 2020 [4–6], and is continuing to increase [7, 8].

Since 2015, a new round of medical policy reform has been implemented in China [9], using the hierarchical diagnosis and treatment system as the means of realizing the rational allocation of resources and promoting the equalization of basic medical and health services. The prevention and treatment of diabetes has been transferred from tertiary hospitals to community health service centers, from simple clinical treatment to comprehensive diagnosis and treatment [5]. In order to establish a standardized management system for diabetes in primary hospitals and improve the treatment and control rate of diabetes, the most important factor is the primary physicians who treat patients with diabetes.

However, a survey found that China’s primary care system performs poorly in diabetes management. Another national longitudinal survey found that diabetes health education coverage has dropped from 76 to 70%. Diabetes examination and treatment methods vary greatly, and the rate of hospitalization and readmission due to diabetes-related diseases has also increased. There was a large gap in the diagnosis and treatment of diabetes, and the rate of hospitalization and readmission for diabetes-related diseases has also increased [10, 11].Continuing medical education has always been regarded as an effective way to improve the capabilities of primary physicians [12, 13].

The aim of the study was to evaluate the effect of primary physician training on standardized management of diabetes, and compare the effects of different training models. Previous research in Liaoning Province showed that the standardized diagnosis and treatment situation is not ideal [14], and 95.5% of primary physicians believe that it is necessary to receive professional training [15]. If the primary physician training can really improve the standardized management of diabetes, then we will further compare the training effects of different training models, and provide reference for optimizing the training contents and forms of different trainees in the future.

## Methods

### Study design

A comparative, pre-post intervention study was used to evaluate the impact of primary physician training. Study subjects consisted of two groups: primary physicians who received training; and patients with diabetes in community health service centers. Using the questionnaire, the short-term training effect on physicians was tested at baseline and at end of training. The long-term training effects on patients were tested at baseline and 1 year after training.

All procedures were implemented in accordance with ethical standards, and all participants were informed of the study purpose, benefits, medical issues, and personal information recorded, and written consent was obtained. The study was approved by the Ethics Committee of China Medical University (Shenyang, China).

### Study population

The study was carried out in Shenyang, Dalian, Jinzhou, and Benxi cities, which were randomly selected in Liaoning Province, China. Six community health service centers were randomly selected from each city and randomly divided into three training groups at a ratio of 1:1:1. The training groups were: (1) Concentrated training group every 6 months—participants received two intensive training sessions on standardized diabetes management in the first and seventh month, each session lasted 4 h; (2) Concentrated training group every 3 months—participants received four intensive training sessions on standardized diabetes management in the first, fourth, seventh, and tenth month, each session lasted 4 h; (3) Short-term intensive training group—participants received eight intensive training sessions on standardized diabetes management knowledge once a week for two consecutive months, each session lasted 4 h. The physician participants in these three groups were 693, 595, and 695, and the patient interviewees were 146, 333, and 106.

Training was carried out in four cities in Liaoning Province in June 2019. Before randomly assigning all community health service centers, baseline data of diabetes management was recorded for almost 12 weeks. After the completion of all training, each community health service center completed a follow-up of the patients included in the baseline within 12 weeks, that is, the questionnaire of all patients was completed before September 2020. Primary physicians completed diabetes management questionnaires before randomization and after training.

This follow-up study was a real-world study. During the implementation of this study, community health service centers were responsible for different areas of patient management, and there were no issues with repeated patient visits. In each community health service center, the training frequency of primary physicians was the same, which reduced the bias caused by patients receiving clinics from different physicians.

### Intervention and data collection

This study aimed to standardize and improve the diagnosis and treatment of patients with diabetes by improving the diabetes management ability of primary physicians. We provided theoretical and practical training for primary physicians through lectures and case studies. The training content of the project was taken from the “*National Guidelines for the Prevention and Management of Primary Diabetes*” (2018 Edition) [16] and “*China’s Guidelines for the Prevention and Treatment of Type 2 Diabetes*” (2017 Edition) [17].

A test questionnaire was designed for physicians based on knowledge related to diabetes diagnosis and treatment, including diagnostic criteria, control goals, treatment plan formulation, and diabetic complication prevention.

Data on patients’ demographic characteristics, family income, living habits, body mass index (BMI), history of disease and medication, and screening of diabetes-related complications were collected by a standardized questionnaire conducted by primary physicians during the patients’ visit. Before the survey was performed, all eligible investigators attended organized training. The training contents included the purpose of this research, how to number the questionnaire, and the importance of standardization.

### Statistical methods

The categorical variables were described in numbers and percentages, and a chi-square test was used for comparing qualitative variables. The continuous variables were summarized as mean and standard deviations (SDs). Logistic regression was used to compare the difference of complication screening rate under different training models. All statistical analyses were implemented in SPSS version 22.0 software and missing data were omitted. The *p* values reported in the study were 2-tailed, and *p* < 0.05 was considered statistically significant.

## Results

### Demographic details of the patients surveyed

A follow-up study of 853 patients with diabetes who underwent a baseline survey was conducted. Data from 585 patients were collected. The loss to follow-up rate was 31.4%. We compared the characteristics of individuals who were and were not lost to follow-up, and found that the course of diabetes was longer and the proportion of men was higher in those who were lost to follow-up (*P* < 0.05).

The characteristics of all the patients are shown in Table 1. The mean age of patients surveyed was 62.7 ± 12.3 (years old), with the largest proportion of patients in the 60–70 age group (46.5%). On average, participants had been diagnosed with diabetes for 12.2 ± 13.7 (years). Mean BMI was 24.8 ± 4.1 (kg/m^2^); nearly 50% were found to be overweight (BMI 25.0–29.9 kg/m^2^) or obese (BMI ≥ 30.0 kg/m^2^), and only 50.6% had a normal BMI. A total of 86.2% of participants were married or living together. Educational level was mainly below middle school (81.8%). The annual family income of most patients was less than 10,000 Yuan (45.8%). Most of the ethnic group was Han (86.4%). Most of the patients with diabetes under investigation had other comorbidities or complications.

**Table 1:** General characteristics of the patient study population (*n* = 853)

Characteristic	Follow-up Group (***n*** = 585)	Lost to follow-up group (***n*** = 268)	***P***-value
Age (mean (SD))	62.7 (12.3)	64.5 (11.3)	0.659
< 30	5 (0.9)	0	
30–40	15 (2.6)	6 (2.2)	
40–50	49 (8.4)	24 (9.0)	
50–60	108 (18.5)	52 (19.4)	
60–70	267 (45.6)	101 (37.7)	
70–80	109 (18.6)	66 (24.6)	
≥ 80	21 (3.6)	17 (6.3)	
Sex			< 0.001
Male	223 (38.1)	140 (52.3)	
Female	362 (61.9)	128 (47.7)	
BMI	24.8 (4.1)	24.3 (3.8)	0.238
< 18.5	14 (2.4)	11 (4.1)	
18.5 ~ 24.9	296 (50.6)	144 (53.7)	
25 ~ 29.9	226 (38.6)	97 (36.2)	
≥ 30	43 (7.4)	13 (4.9)	
Diabetes course, years	12.2 (13.7)	14.7 (16.1)	0.003
Marital			0.206
Married or living with partner,	502 (85.8)	221 (82.5)	
Unmarried, divorced or widowed	81 (13.8)	46 (17.1)	
Education level			0.942
Primary school or below	249 (42.6)	116 (43.3)	
Middle school	229 (39.1)	101 (37.7)	
High school and above	106 (18.1)	49 (18.3)	
Annual family income (CNY/year)			0.969
< 10,000	264 (45.1)	118 (44.0)	
10,000-30,000	183 (31.3)	89 (33.2)	
30,000-50,000	72 (12.3)	33 (12.3)	
≧50,000	58 (10.0)	27 (10.1)	
Ethnicity			0.832
Han	505 (86.3)	226 (84.3)	
Manchu	55 (9.4)	29 (10.8)	
Hui	4 (0.7)	3 (1.2)	
Mongolian	19 (3.2)	10 (3.7)	
Others	1 (0.2)	0	
OAD	456 (77.9)	208 (77.6)	0.543
Metformin	393 (86.2)	180 (86.5)	
Insulin secretagogues	115 (25.2)	53 (25.5)	
α-glucosidase inhibitors	62 (13.6)	27 (13.0)	
DPP4 inhibitors	1 (0.2)	0	
SGLT2 inhibitors	2 (0.4)	2 (1.0)	
Insulin therapy	201 (34.4)	94 (35.1)	0.547
Premixed human insulin 30R	88 (24.9)	33 (35.1)	
Premixed insulin analog 30 or 25	30 (9.4)	15 (16.0)	
Premixed human insulin 50R	16 (5.2)	11 (11.7)	
Premixed insulin analog 50	8 (2.6)	1 (1.1)	
Basal insulin	46 (4.2)	28 (29.8)	
Mealtime insulin	31 (10.0)	19 (20.2)	
GLP-1 receptor agonist	1 (0.3)	0	
Dual combination therapy	109 (18.6)	57 (27.4)	0.808

*OAD* Oral antidiabetic drugs, *DPP4* Dipeptidyl peptidase 4, *SGLT2* Sodium-dependent glucose transporters 2, *Premixed human insulin 30R* 30% regular insulin and 70% NPH insulin, *Premixed human insulin 50R* 50% regular insulin and 50% NPH insulin, *GLP-1* Glucagon-like peptide-1

Metformin is the most commonly used oral anti-diabetic drug (78.9%), and the use rate of human premixed insulin 30R (18.2%) is the highest in insulin therapy. A total of 19.3% of patients received dual therapy with oral medication and insulin therapy.

### The short-term training effect of physicians

**Fig. 1:**
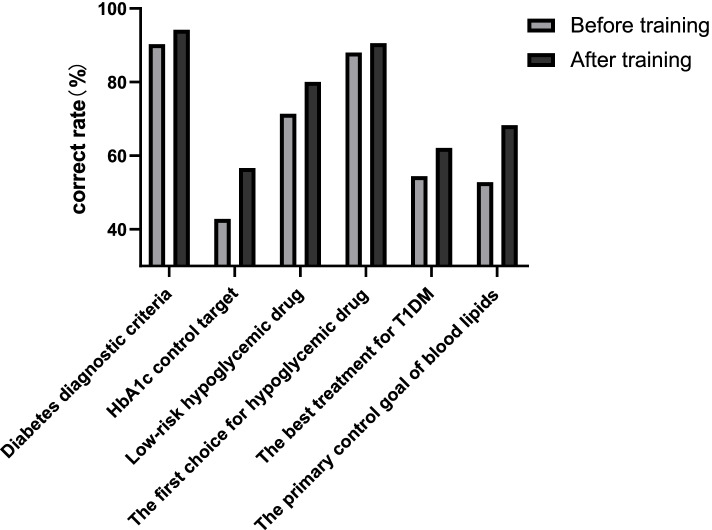
Correctness rate of primary physicians before and after training

A total of 2083 primary physicians participated in the training and submitted the questionnaire. The correct rate of answering questions on standardized diabetes management is shown in Fig. 1. After training, diabetes management knowledge of primary physicians improved (*p* < 0.05).

### The long-term training effect of patients

As shown in Table 2, the complication screening rate of local diabetes patients increased from 22.2% before training to 27.7% 1 year after training (*p* = 0.033). The medication rate of patients with diabetes, hypertension, and hyperlipidemia was higher than before the training (*p* < 0.05).

**Table 2:** Diabetes management before and after training

Parameter	Before Training	After Training	***P*** value
Metformin use	350 (72.2)	393 (78.6)	0.019
Insulin therapy	201 (48.9)	307 (64.6)	< 0.001
Hypotensive drug use	286 (82.2)	311 (89.4)	0.007
lipid-lowering drug use	91 (40.6)	148 (79.1)	< 0.001
HbA1c testing	105 (21.0)	120 (21.0)	0.995
Complication screening (total)	130 (22.2)	155 (27.7)	0.033
Diabetic fundus disease screening	102 (19.7)	108 (19.7)	0.994
Diabetic nephropathy screening	85 (16.4)	117 (20.9)	0.060
Diabetic neuropathy screening	63 (12.2)	97 (17.4)	0.017
Diabetic vascular disease screening	68 (13.2)	124 (22.2)	< 0.001

### Differences in training models

The complication screening rate of the three groups after training was 27.8, 24.6, and 37.6% respectively. There was a statistically significant difference (*p* < 0.05) between complication screenings in the three training groups. The pairwise comparison was statistically different between the concentrated training group at 3-month intervals and the short-term intensive training (*p* = 0.011, *p* < 0.0125). Table 3 shows the results of univariate and multivariable logistic regression for complication screening. It can be seen that the short-term intensive training group (OR = 1.806, 95%CI 1.008–3.233) and younger people (OR = 0.972, 95%CI 0.949–0.996) are more likely to undergo complications screening.

**Table 3:** Logistic regression analyses to determine the influencing factors of complication screening

Characteristics	Univariate OR (95%CI)	Multivariable OR (95% CI)
Training model		
Concentrated training group every 6 months	1	1
Concentrated training group every 3 months	1.568 (0.895,2.747)	1.407 (0.696,2.844)
Short-term intensive training group	1.846 (1.150,2.964)	1.806 (1.008,3.233)
Age	0.982 (0.964,1.000)	0.972 (0.949,0.996)
Sex		
Male	1	1
Female	0.768 (0.527,1.121)	0.743 (0.455,1.212)
BMI	1.020 (0.966,1.077)	0.996 (0.929,1.067)
Courses	0.998 (0.980,1.017)	0.996 (0.977,1.016)

*Courses* Course of diabetes mellitus

## Discussion

The results indicated that training is an effective way to improve primary physicians’ knowledge about diabetes and enhancing standardized diabetes management in hospitals. In the short-term training group, physicians improved their knowledge of diabetes diagnosis and treatment after training. In terms of long-term results, the primary physicians’ transition to clinical practice from theoretical knowledge improved the standardized management of patients with diabetes.

After training, the knowledge scores of participants improved considerably. This suggested that training can effectively improve primary physicians’ knowledge about diabetes standardized management, which is consistent with the results of previous studies [18–20]. The survey of participants indicated that their understanding of all aspects of diabetes improved with training. A diagnosis of diabetes is easy to understand in a short time, however, other aspects of disease management need long-term clinical practice to strengthen and consolidate. The participation of primary physicians in standardized training can enrich their knowledge reserves, provide vital decisions to improve the prognosis of patients, and ensure the systematic and continuity of care in the health care system [10].

Previous studies have showed that primary physicians with relevant experience in diabetes diagnosis and treatment delivered higher-quality care [21]. This indicates that primary physicians who receive training can improve the quality of diagnosis and treatment of patients. However, if a training strategy is effective, the effect should be long lasting. The follow-up after one-year was used to evaluate the long-term effect of the primary physician training. During the 3 months post-training, patients with diabetes who participated in the baseline survey were followed up, and results indicated that the standardized medication and complication screening of interviewees had improved. However, the rate of patients lost to follow-up in this study is relatively high (31.4%). Compared with the non-lost-to-follow-up population, the lost-to-follow-up population had a larger proportion of patients with longer duration of diabetes and > 70 years of age, which made it more difficult for patients to receive telephone follow-up, and also increased the probability of adverse outcomes and death, thus reducing the follow-up rate. In addition, the proportion of men was significantly higher than that of women, which may be related to different behaviors, lifestyles or stress, or attitudes towards treatments and prevention between women and men.

Chronic complications of diabetes and related diseases are significant causes of disability or death of patients. The guidelines suggest that clinicians should ensure appropriate screening for complications and comorbidities among patients with type 1 at 5 years after diagnosis, and all Type 2 diabetes patients should undergo annual diabetic nephropathy screening and annual comprehensive eye examination [22]. We noticed that the average course of the disease in patients was 12.2 ± 13.7 years, but the screening rates of diabetic complications in primary hospitals were very low. This study shows that after training, the screening rate of patients with various complications significantly improved. As it is inevitable that the prevalence of complications will increase, early complication screening can help to identify complications earlier, improve the patients’ quality of life, and improve the patients’ prognosis. Therefore, standardized management of diabetes should be promoted and implemented on a large scale.

Primary physicians are a key component of health care delivery in many countries in the world, but in China, there has been a transition from barefoot doctors to primary health providers [23]. With the rapid economic development, China has sufficient reserves to standardize diabetes management in the primary health care system. However, in the real world, great gaps in the quality of primary health care still exist, and the continuing medical education and training of primary physicians is an effective way to resolve this gap. We need to identify the most suitable training model according to local conditions. In this study, we designed three different training models and found that the model of short-term intensive training worked best. After short-term intensive training, the screening rate of complications increased by 14.0%. The results of multivariate logistic regression showed that the probability of complication screening in the short-term intensive group was 1.806 times higher than that in the concentrated training group every 6 months in the whole year.

This is the first study to conduct standardized diabetes management training for primary physicians in northeast China. We considered the impact of training on both physicians and patients, clarified the significance of primary physician training for clinical practice, and explored the differences between training models. While the different characteristics of training will affect health care workers in different ways [24], improvement strategies need to consider the differences between clinical goals and consider tailored methods instead of a “one size fits all” method [25]. Future research should explore training models on the basis of this research, aiming to find the most suitable model for the local conditions in Liaoning Province.

Despite the above findings, this study still has some limitations. First, we selected only 24 community health centers from four cities in Liaoning Province, which may restrict us from extending these findings to other areas. Second, the study lacks a control group that did not receive training during the same period. Therefore, we need to interpret observed changes to our intervention carefully. Additionally, due to the pandemic in 2020, the difficulty of data collection increased. During 2020, the number of patients with diabetes attending primary medical institutions decreased, which may limit the training effect of this study.

## Conclusion

In conclusion, our study evaluated the effect of a primary physician training on the standardized management of diabetes. The diabetes-related knowledge of primary physicians, and the diagnosis and treatment situation of patients, both improved. However, the training effect was different in primary physicians with different training models. Therefore, when conducting primary physician training, the training model should be adjusted according to the specific conditions of each location to achieve the best training effect.

## Supplementary Information

**Additional file 1.**

**Additional file 2.**

## Data Availability

The datasets used and/or analyzed during the current study are available from the corresponding author on reasonable request.

## Abbreviations

BMI: Body mass index
SDs: Standard deviations
OAD: Oral antidiabetic drugs;
DPP4: Dipeptidyl peptidase 4
SGLT2: Sodium-dependent glucose transporters 2
GLP-1: Glucagon-like peptide-1
