# Supplementary material for: The role of primary physician training in improving regional standardized management of diabetes: a pre-post intervention study
Source: BMC Prim Care. 2022 Mar 21;23:51. doi: 10.1186/s12875-022-01663-5 (PMC8939124; doi:10.1186/s12875-022-01663-5)
Supplement: Supplementary file 1 — Additional file 1. [file 12875_2022_1663_MOESM1_ESM.docx]

**Additional file 1**

**1. Diabetes Knowledge Questionnaire**

| 1. What are the diagnostic criteria for diabetes? | Fasting blood glucose≥6mmol/L, 2 hours postprandial blood glucose≥7.8mmol/L |
| --- | --- |
|  | Fasting blood glucose ≥7mmol/L, 2 hours postprandial blood glucose ≥11.1mmol/L |
| 1. What is the goal of glycated hemoglobin control in the general population of diabetic patients? | HbA1c<7% |
|  | HbA1c<8% |
|  | HbA1c<6.5% |
|  | HbA1c<6% |
| 1. Which of the hypoglycemic drugs has a higher risk of hypoglycemia? | Metformin |
|  | Sulfonylureas |
|  | Glycosidase inhibitor |
|  | DPP4 inhibitor |
| 1. If there are no contraindications or gastrointestinal adverse reactions, which type of drug should be preferred for patients with type 2 diabetes? | Metformin |
|  | Sulfonylureas |
|  | Glycosidase inhibitor |
|  | DPP4 inhibitor |
| 1. What is the best treatment plan for patients with type 1 diabetes? | Two or three injections of pre-mixed insulin analogs daily |
|  | One super long, three super short or insulin subcutaneous pump |
|  | Two injections of human premixed insulin daily |
|  | Two or three injections of short-acting insulin daily |
| 1. What is the primary control goal of blood lipids in diabetic patients? | LDL-C<2.6mmol/L |
|  | TG<1.7mmol/L |
|  | TC<4.5mmol/L |
|  | HDL-C>1.0mmol/L |

**2. Diabetes questionnaire**

Part one. General Situation

A1. Name:

A2. Gender: Male / Female

A3. Date of birth:

A4. Identification number:

A5. Address:

A6. Phone number:

A7. How many people are there in your family?

A8. What is your education level?

1. Primary school or below 2. Middle school 3. High school and above

A9. What is your current marital status?

1. Married or living with partner 2. Unmarried, divorced or widowed

A10. What is your nationality?

1. Han 2. Manchu 3.HuI 4.Mongolian 5.Others

A11. Last year, what was the total per capita income (including various sources) of your family for the whole year?

1. <10,000 2. 10,000-30,000 3. 30,000-50,000 4. ≧50,000

A12. Your current height is cm

A13. Your current weight is kilograms

A14. What is your blood type?

1. Type A 2. Type B 3.Type O 4.Type AB 5.Don't know

Part two. Disease history and medication history

B1. Have you ever been diagnosed with the following diseases by a doctor?

| Disease name | Whether you have the disease | | Age at first diagnosis |
| --- | --- | --- | --- |
|  | Yes | No |  |
| Diabetes | 1 | 2 |  |
| Dyslipidemia | 1 | 2 |  |
| Hypertension | 1 | 2 |  |
| Diabetic nephropathy | 1 | 2 |  |
| Diabetic retinopathy | 1 | 2 |  |
| Diabetic peripheral neuropathy | 1 | 2 |  |
| Coronary heart disease | 1 | 2 |  |
| Stroke | 1 | 2 |  |

B2. Are you currently using the following drugs?

| Drug name | Yes | No | How many months have been taken |
| --- | --- | --- | --- |
| Metformin | 1 | 2 |  |
| Acarbose | 1 | 2 |  |
| DPP4 inhibitor | 1 | 2 |  |
| Sulfonylureas | 1 | 2 |  |
| Glinide | 1 | 2 |  |
| SGLT2 inhibitor | 1 | 2 |  |
| Premixed human insulin 30R | 1 | 2 |  |
| Premixed insulin analog 30 or 25 | 1 | 2 |  |
| Premixed human insulin 50R | 1 | 2 |  |
| premixed insulin analog 50 | 1 | 2 |  |
| basal insulin | 1 | 2 |  |
| Meal insulin | 1 | 2 |  |
| Liraglutide or Exenatide | 1 | 2 |  |
| Aspirin drugs | 1 | 2 |  |
| Lipid-lowering drugs | 1 | 2 |  |
| Antihypertensive drugs | 1 | 2 |  |

Part three: Weight, blood pressure, blood sugar, blood lipids and other information

C1. In the past 12 months, have the doctors of the primary medical institution provided you with any examinations or guidance?

| Items | Yes | No |
| --- | --- | --- |
| Blood pressure measurement | 1 | 2 |
| Blood lipid measurement | 1 | 2 |
| Weight control | 1 | 2 |
| Quit or smoke less | 1 | 2 |
| Quit or drink less alcohol | 1 | 2 |
| Diabetic Fundus Disease Screening | 1 | 2 |
| Diabetic nephropathy screening | 1 | 2 |
| Peripheral neuropathy screening | 1 | 2 |
| Vascular arteriosclerosis screening | 1 | 2 |

C2. Blood sugar and its control

| How long has it been since you last measured your blood sugar? (Including self-test at home and going to medical institutions for testing.) | 1. Within 6 months  2. Within 12 months  3. Within 2 years  4. 2 years ago  5. Never tested blood sugar |
| --- | --- |
| Before participating in this survey, did you know your blood sugar level? | 1. Above the normal range  2. It belongs to the normal range  3. Below the normal range  4. Don't know |
| Detection of glycosylated hemoglobin within half a year | 1. Yes the value is % 2. No |

**Thank you very much for your support and cooperation!**
