# Supplementary figures and images for: The role of primary physician training in improving regional standardized management of diabetes: a pre-post intervention study
Source: BMC Prim Care. 2022 Mar 21;23:51. doi: 10.1186/s12875-022-01663-5 (PMC8939124; doi:10.1186/s12875-022-01663-5)

Flow diagram：


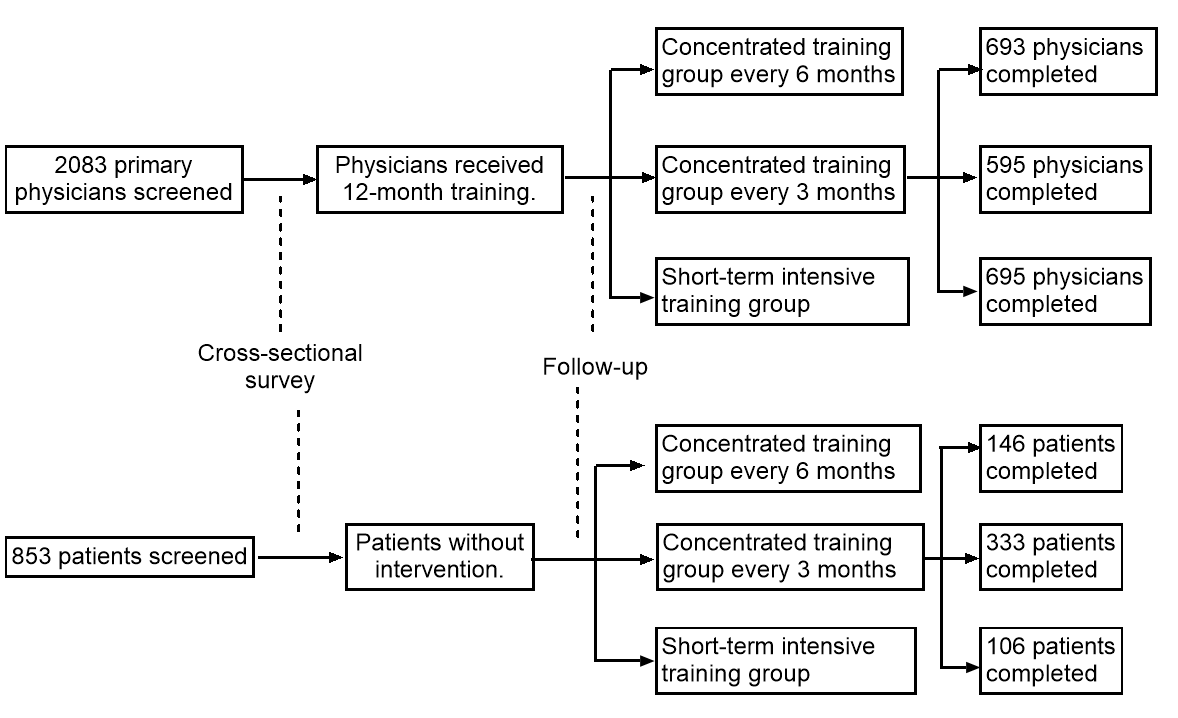

Supplement: Supplementary file 2 — Additional file 2. [file 12875_2022_1663_MOESM2_ESM.docx]
